# Connecting the beholder with the artwork: Thoughts on gaining liveliness by the usage of paraphernalia

**DOI:** 10.1177/20416695231162010

**Published:** 2023-03-09

**Authors:** Claus-Christian Carbon

**Affiliations:** Department of General Psychology and Methodology, University of Bamberg, Bavaria, Germany;; Research Group EPAEG (Ergonomics, Psychological Aesthetics, Gestalt), Bamberg, Germany;; Bamberg Graduate School of Affective and Cognitive Sciences (BaGrACS), Bamberg, Germany;

**Keywords:** perception, empirical aesthetics, sculptures, paraphernalia, face masks, sunglasses, gestalt, vividness, like-alikeness, vivid eyes

## Abstract

When we attend sculptures in museums, they might fascinate us due to the mastery of the material, the inherent dynamics of body language or due to *contrapposto* or the sheer size of some of these statues such as Michelangelo's David. What is less convincing, however, is the life-alikeness of the face. Actually, most visitors experience dead faces, dead eyes, and static expressions. By merely adding paraphernalia to a face (e.g., a facemask or sunglasses), such unalive sculptures gain vividness and liveliness. This striking effect is demonstrated by applying a facemask and sunglasses to a sculpture on public display in Bamberg, but it can easily be demonstrated on any available sculpture. This simple method might help connect people with sculptures or artworks, in general, to lower the barrier between the beholder and artwork and increase their interaction.

Human sculptures often appear dead—with some exceptions, for instance, wax figures that are mainly created to shock, provocate, or even horrify the beholders. That is no surprise; actually, sculptures are not alive. Artworks, however, do not have to be alive to appear vivid (instead of dead) and invite their beholders to connect with them. A lot of human sculptures throughout art history were created to provide a vivid representation or reference to living persons—for instance, to a beloved one, like in the case of Antinous, who probably was the favourite lover of the Roman emperor Hadrian (see Figure 1a and b). Artists have developed sophisticated techniques to make lively characters and to enable or even deepen the connection between artwork and the beholder for hundreds of years. One important method in this respect was to drill a little hole or etch circles in sculptured eyeballs to create the illusion of a pupil, giving a more lively impression (Figure 1b). But this technique also bears the risk of creating a staring expression, as often attributed to Michelangelo's *Proculus* (1494−1495) and as Figure 1b and c indicates. Using eye inlays made of marble and glass, as realized in many Greek and Roman Imperial statues (Steiner, 2001), allows for greater realism. Yet, the results often remain unconvincing since the material fixation of the eyes’ position and the gaze bring about mostly static expressions.

**Figure 1.**
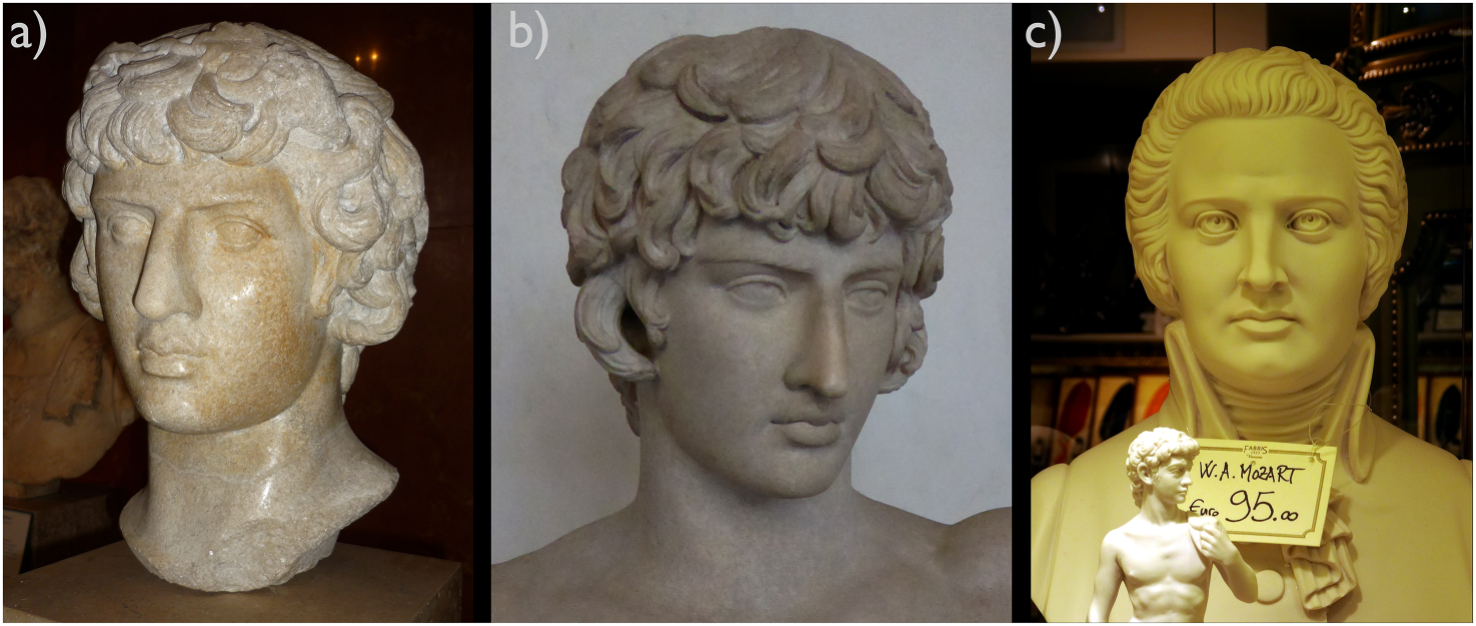
(a) Roman marble bust of Antinous as Dionysus from about 130 CE. Louvre Museum in Paris, France. (b) Roman marble bust of Antinous from 130 to 138 CE, Galleria degli Uffizi Florence, Italy. (c) Commercial plaster bust of W. A. Mozart on the display of a tourist shop in Vicenza, Italy. Photos were taken by CCC in October 2010, March 2015, and June 2014, respectively.

So, how to achieve an inviting impression of activity and vividness in a static object? Typical techniques for depicting motion in paintings, such as blur, action lines, or arrows (Cutting, 2002), are not easily applicable in sculptural works. The well-known method to impose vividness in human sculptures via *contrapposto* might work for larger movements of the body's extremities (Pazhoohi et al., 2020), but not for the face. Another attempt to render human sculptures more vivid and approachable that was already used in ancient times is polychromy (Brinkmann, 2008). Today's recipients partly show a rejective attitude towards polychrome ancient sculpture, especially if they are not sufficiently familiarized with such versions (cf. Carbon & Hesslinger, 2014). On the other hand, there are also works that are appreciated primarily for their vivid polychromy such as the hyperrealistic sculptures by Duane Hanson (1925–1996) which the artist created from casts of actual individuals using different materials. Hansons's approach is, of course, very different from that of most classical sculpturors who stuck to a single kind of material and a singular sculptural technique (Duby & Daval, 2010).

There is a very simple option that probably works for portraits as well, but is especially effective for sculptures. I found this simple method accidentally when visiting a sculptural work in Bamberg—and it seems there were others who played with that already, for example, sculptor Isa Genzken equipped copies of the Nefertiti with paraphernalia in her 2015 *Nefertiti series*. The artwork I attended in 2020 during the COVID-19 pandemic consists of eight bronze casts that are sitting in a circle (see Figure 2a). Although the whole group as a Gestalt makes quite a vivid impression, the single characters lack liveliness (Figure 2b).

**Figure 2.**
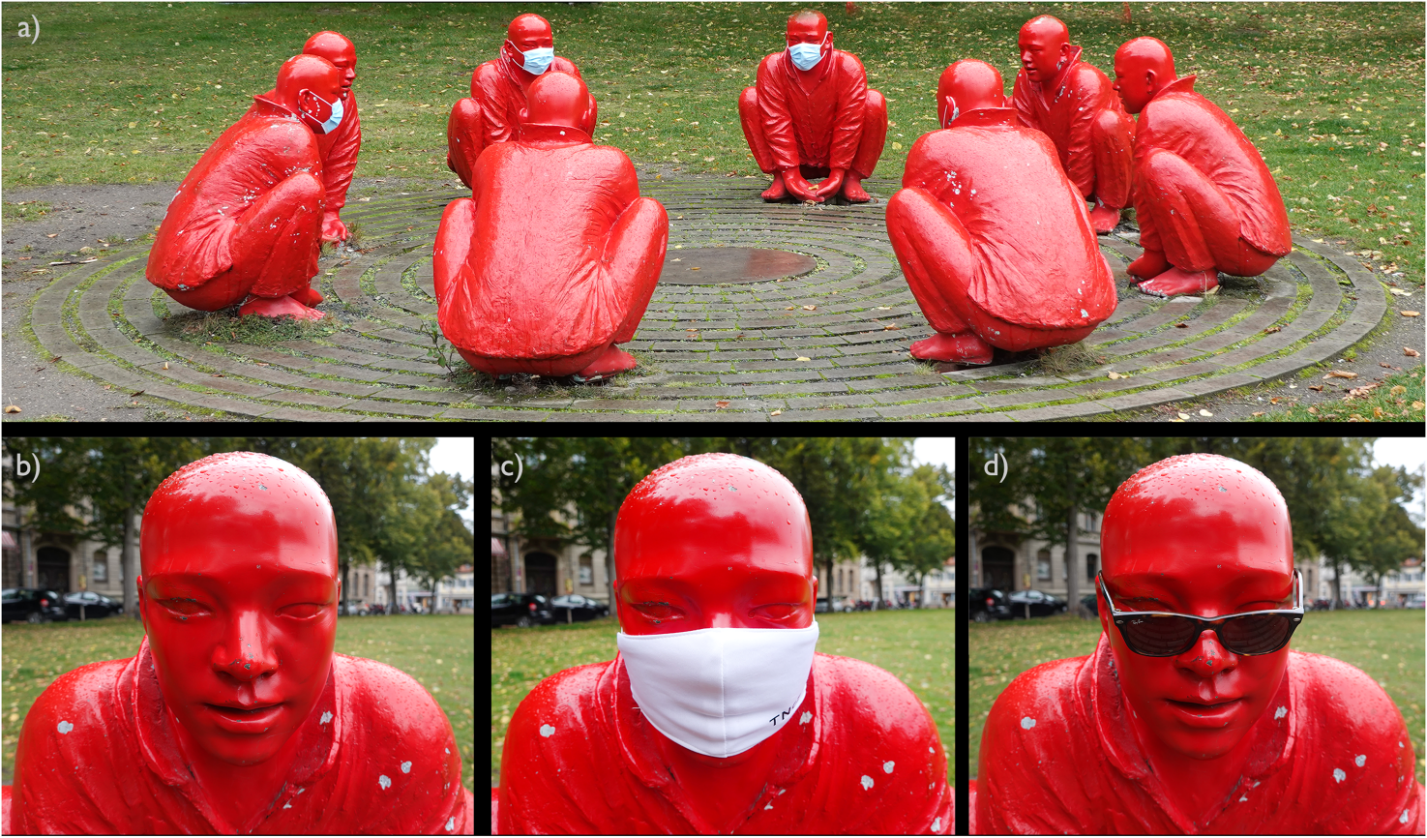
(a) This is the group of sculptures called “meeting” on Schönleinsplatz, Bamberg/Bavaria, which was created by Chinese artist Wang Shugang for an exhibition in 2013. The group consists of eight red-painted bronze casts. Important note: the figures were originally not equipped with face masks but were temporarily covered by them as a spontaneous reaction to the COVID-19 pandemic. (b) Part of the original sculpture, (c) temporarily equipped by the author with a face mask, and (d) sunglasses, respectively. Photos were taken by CCC in October 2020.

As a reaction to the anti-COVID measures that demanded the wearing of face masks in 2020, visitors to the sculpture equipped some of the bronze casts with surgical masks which drew my attention (Figure 2a). This little addition had a remarkable effect—actually, heads with a facemask appeared much more vivid and “real,” and when I played around equipping one figure selectively with a face mask (Figure 2c) and with sunglasses (Figure 2d) such simple additions increased the life-alikeness substantially. What happened due to this intervention? Covering parts of the facial information seems to have triggered perceptual completion that resulted in an imaginary Gestalt compatible with a very vivid proxy of a real human. Imagination obviously does not create a lifeless object but a living creature.

**Empirical evidence**. To test whether this first impression of more liveliness in artworks by equipping them with unusual paraphernalia is empirically observable, we conducted an online study (*N *= 35, based on an a priori power test with a proposed effect size of *f *= 0.25, and set alpha = .05 and beta = .20 yielding a minimum sample size of 28, which we overpowered by 20% to cope with potential data loss; we gained an actual power of .81) with four different sculptures (Socrates, Plato, Aristotle, and Red Sculpture—see Figure 2b) with four different presentation conditions (*none*, original; *SunG*, sunglasses; *Mask*, facemask; *Mask&SunG*, facemask and sunglasses together) each. Each participant was exposed to all 4 [sculptures] × 4 [presentation conditions] versions, one by another, in a fully randomized order. To cover a broader spectrum of relevant variables of aesthetic experience, we used three different measures that were asked for each version in the following order: (a) how lively does the sculpture appear, (b) how thought-provoking is the sculpture, and (c) how interesting is the respective sculpture? The mean total length of the study was 7.5 min.

As shown in Figure 3, we detected the strongest effects on liveliness when applying sunglasses; applying a face mask plus sunglasses was the most thought-provoking, but only for the classic sculptures of Plato and Aristotle. Applying a face mask had apparent positive effects on the variables lively and thought-provoking.

**Figure 3.**
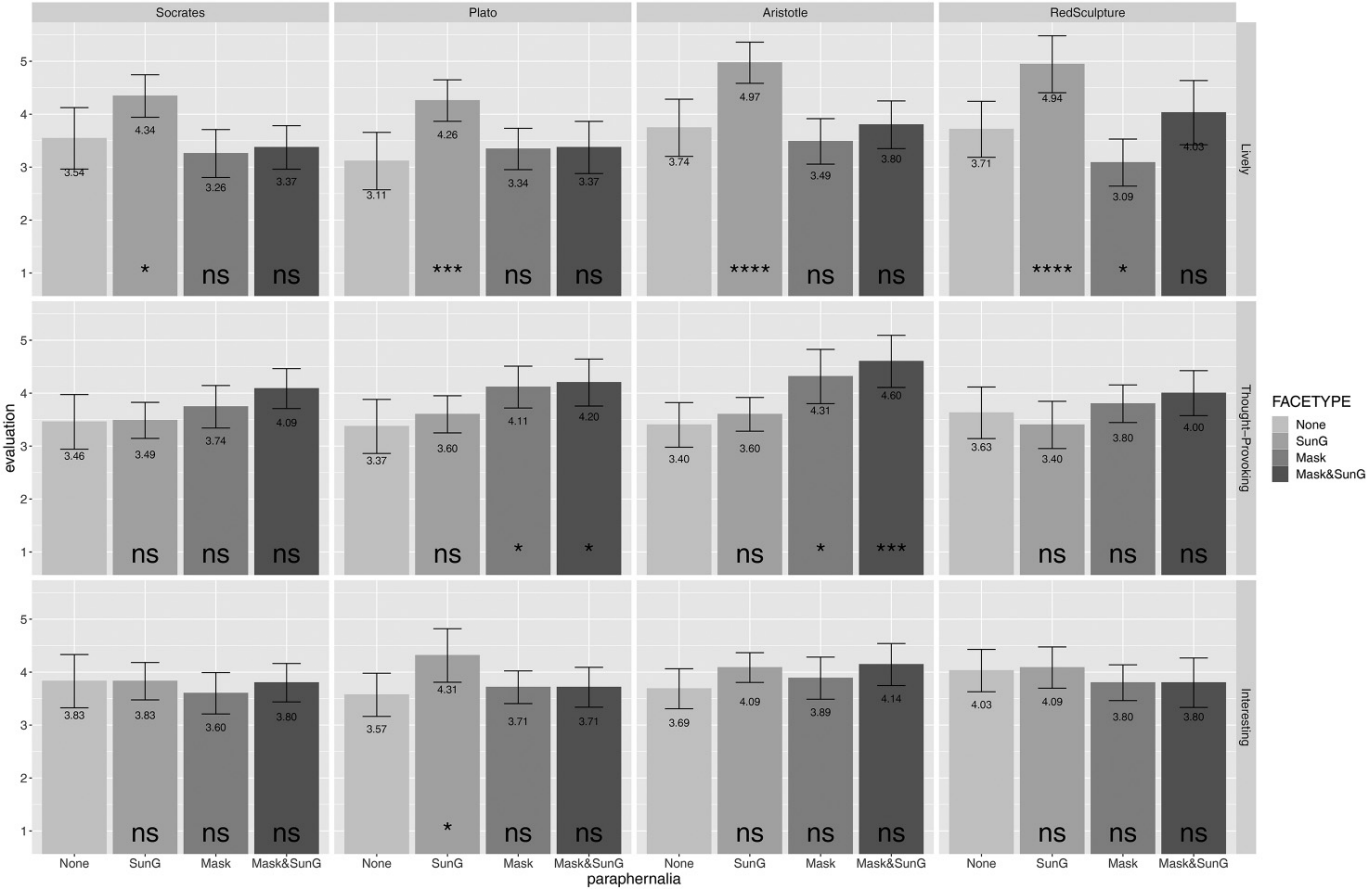
Results for four different sculptures (Socrates, Plato, Aristotle, and Red Sculpture) with four different presentation conditions (*none*, original; *SungG*, sunglasses; *Mask*, facemask; *Mask&SunG*, facemask and sunglasses together) each. The rows show the results for the three dependent variables, that is, lively, thought-provoking, and interesting. *p < .05, ***p < .001, ****p < .0001, based on *t*-tests comparing conditions with the *none*-condition.

**Coda**. We can easily give dead sculptures back their life (sculptures were unalive all their life, true, but at least at the beginning of their career, the artist breathed life into the unaddressed chunk of raw material, whether marble, plaster, or wood). The effect is appealing, and artworks emerge more lively and thought-provoking; similar effects are intuitively used by kids equipping their snowmen with scarves, branches, and carrots. This mixture might change our perspective and probably invite us to rethink our established evaluations and associations with artworks of all kinds—what an excellent opportunity to have more vivid and engaged interaction with artworks.
